# Associations between the oxidative balance score and constipation: a cross-sectional study of the NHANES, 2005–2010

**DOI:** 10.1186/s12889-024-19428-3

**Published:** 2024-07-16

**Authors:** Yuchao Wang, Jiao Li, Wei Sun, Yunbin Tong, Lu Han, Ziying Jiang, Weili Xu, Daqing Sun

**Affiliations:** 1 Department of Pediatric Surgery, Tianjin Medical University General Hospital, Tianjin, 300052 China; 2 Department of General Surgery, Tianjin Haihe Hospital, Tianjin, 300350 China; 3 Department of Pediatric Surgery, The Second Hospital of Hebei Medical University, Shijiazhuang, 050000 China

**Keywords:** Oxidative Balance Score, Constipation, Oxidative Stress, NHANES

## Abstract

**Objective:**

The oxidative balance score (OBS) reflects the overall burden of oxidative stress in an individual, with a higher OBS indicating greater antioxidant exposure. This study aimed to explore the association between constipation and OBS.

**Methods:**

Variables were extracted from participants who completed a constipation questionnaire as part of the National Health and Nutrition Examination Survey (NHANES) from 2005 to 2010. The OBS was developed based on dietary and lifestyle factors, encompassing 16 nutrients and 4 lifestyle variables. Weighted logistic regression and restricted cubic spline (RCS) analyses were employed to evaluate the association between OBS and constipation.

**Results:**

After adjusting for all covariates, weighted multivariate logistic regression analysis revealed a 4% reduction in the incidence of constipation for each additional unit of OBS (OR: 0.96, 95% CI: 0.95–0.97, *p* < 0.001). In the OBS subgroup, the risk of constipation significantly decreased compared to that in the lowest quartile (Q2: 0.72, *P* = 0.024; Q3: 0.59, *P* < 0.001; Q4: 0.54, *P* < 0.001).

**Conclusions:**

The present study demonstrated a significant association between constipation and the oxidative balance score (OBS), particularly dietary OBS, and that an increase in OBS may reduce the risk of developing constipation, in which oxidative stress may play an important role. This finding suggested that dietary modification could be an important approach for preventing constipation.

**Supplementary Information:**

The online version contains supplementary material available at 10.1186/s12889-024-19428-3.

## Introduction

Constipation, characterized by a low frequency of defecation, difficulty in defecation, and incomplete defecation, significantly impacts quality of life [1]. The global incidence of constipation is on the rise, attributed to shifts in dietary habits and lifestyles, with an approximately 15% prevalence worldwide, which is higher among adults than among children [2]. The prevalence of this condition increases with age, with females being more prone to chronic constipation than males are [3], which seriously affects patients’ quality of life and physical and mental health. Currently, pharmacological treatment and microbiological therapies remains the mainstay treatment for constipation. However, mainstream pharmacological treatment, such as various laxatives and pro-biotic and pro-secretory drugs, usually fail or have only short-term efficacy and may cause side effects [4]. Only approximately 22% of patients with constipation seek medical care, and dissatisfaction with current treatments underscores the need for alternative preventive approaches addressing underlying causes.

Currently, numerous studies have confirmed that elevated levels of oxidative stress are observed in patients with constipation and animal models, suggesting a potential link between constipation and oxidative stress [5], which may be related to the elevation of NADPH oxidase in patients with chronic constipation and the fact that oxidative stress can further affect intestinal neurons [6, 7].The oxidative balance score (OBS) serves as a metric for antioxidant and pro-oxidant exposure in diet and lifestyle, reflecting overall oxidative stress levels [8]. OBS has been validated in several studies involving diabetes, Non-alcoholic fatty liver disease, and periodontitis [9–11]. The OBS has the potential to assess the impact of lifestyle and dietary factors on oxidative stress. OBS can assess the impact of lifestyle and dietary factors on oxidative stress. Among the dietary indicators in the OBS, dietary fiber, β-carotene, vitamin B2, niacin, vitamin B6, total folate, vitamin B12, vitamin C, vitamin E, calcium, magnesium, zinc, copper, and selenium, most of which have been shown to affect intestinal health and lead to changes in bowel habits. There are no studies confirming the relationship between OBS and constipation; therefore, the aim of this study was to assess the relationship between OBS and constipation through a cross-sectional analysis.

## Materials and methods

### Study population

**Fig. 1:**
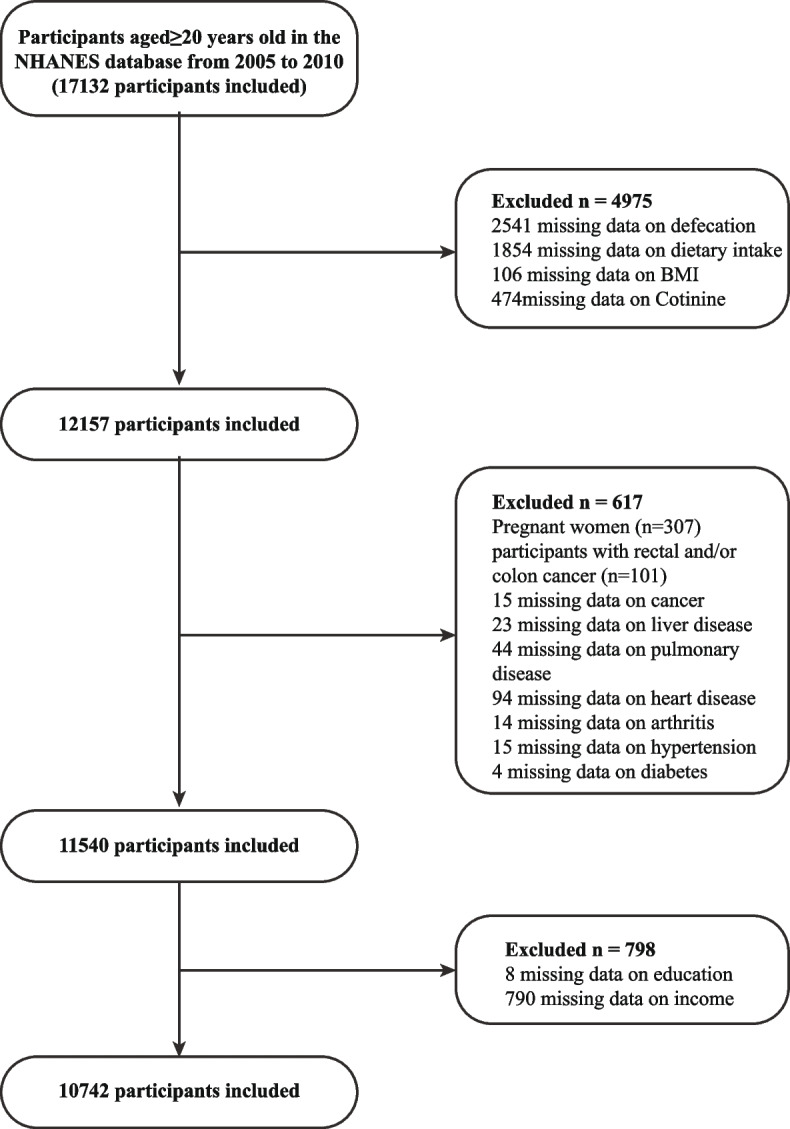
Study population screening flow chart

The National Health and Nutrition Examination Survey (NHANES) database, led by the National Center for Health Statistics (NCHS), assesses the health and nutritional status of U.S. adults and children. It employs a complex, multistage probability sampling design, annually surveying approximately 5,000 participants. Administered by the Centers for Disease Control and Prevention, the NHANES ensures a representative sample of the total U.S. population through random selection. For this study, data from the 2005–2006, 2007–2008, and 2009–2010 surveys were integrated due to their comprehensive gut health questionnaires [12]. Only adults aged ≥ 20 years were included, with participants excluded if they had rectal and/or colon cancer or were pregnant. [13] Additionally, participants with missing covariate data were excluded. Ultimately, 10,742 participants were included in the analyses, as illustrated in Fig. 1.

### Constipation

The NHANES employs bowel frequency and stool type to assess constipation in participants completing the Bowel Health Questionnaire [14]. During the survey, participants estimated their weekly defecation frequency. According to previous NHANES data, less than 3 defecation per week indicates constipation, 3–21 defecation per week are considered normal, and more than 21 movements per week suggest diarrhea. Additionally, participants are classified as constipated if they report type 1 (hard lumps) or type 2 (sausage-like with lumps) stools based on the Bristol Stool Classification. [15].

### Oxidative balance score (exposure)

The oxidative balance score (OBS) was developed based on dietary and lifestyle factors, incorporating 16 nutrients and 4 lifestyle variables. Dietary intake data including nutrients such as dietary fiber, carotenoids, riboflavin, etc. were assessed for each subject by Dietary Interview—Total Nutrient Intakes from two 24-h dietary recall interviews. It was also analyzed based on the average intake over two days [16]. Lifestyle factors such as physical activity, body mass index (BMI), alcohol consumption, and smoking status were also considered, with smoking intensity measured by cotinine levels. Among these factors, total fat, iron, BMI, alcohol consumption, and smoking were categorized as pro-oxidants, while the remaining nutrients were classified as antioxidants [17]. Each component of diet and lifestyle was assigned a score based on its antioxidant or pro-oxidant properties, as well as gender (male or female), following the allocation scheme of the OBS components. The overall OBS score was calculated as the aggregate of individual component scores. A higher OBS signifies greater antioxidant exposure. Following the method proposed by Zhang et al. for calculating OBS, participants in this study were categorized into three groups based on their alcohol consumption: heavy drinkers (≥ 15 g/day for women and ≥ 30 g/day for men), moderate drinkers (0—15 g/day for women and 0—30 g/day for men), and non-drinkers. The participants were assigned scores of 0, 1, and 2, respectively [18]. The remaining components were categorized into three tiers based on sex. In the antioxidant group, the scores ranged from 1 to 3, with position 1 receiving a score of 2 and position 3 receiving a score of 0; conversely, in the pro-oxidant group, position 1 was assigned a score of 0, and position 3 was assigned a score of 2.

### Covariate

Given the potential confounding of findings by other factors, we incorporated covariates into our analyses, as informed by prior research [19]. Using data from NHANES interviews, laboratory tests, and questionnaires, we integrated covariates such as age, sex, race, educational level, poverty-to-income ratio (PIR), body mass index (BMI), and various health conditions, including hypertension, diabetes, cardiovascular disease (CVD), arthritis, and cancer. Socioeconomic variables were evaluated and documented through household interviews. The poverty-to-income ratio (PIR) was stratified into < 2 and ≥ 2 groups according to the original survey data, with lower PIR values correlating with increased poverty levels. The participants’ behavioral characteristics were self-reported. Body mass index (BMI) was assessed according to MEC standards and grouped into three categories: < 25, 'under/normal weight'; 25–30, 'overweight'; and ≥ 30, 'obese'. Hypertension, diabetes, arthritis, liver disease, and cancer status were ascertained through self-reports of having been diagnosed with these conditions. Individuals who were diagnosed with congestive heart failure, coronary heart disease, heart attack, or angina pectoris were categorized as having heart disease, while those with asthma, chronic bronchitis, or pulmonary emphysema were classified as having lung disease.

### Statistical analysis

Given the intricate nature of the national population sampling design, we incorporated 2-year dietary weights (WTDR2D), sampling units (SDMVPSU), and strata (SDMVSTRA) in all our analyses. Descriptive analyses were conducted for all participants. Continuous data for all participants were analysed using means and standard deviations (SDs) based on the data type. Categorical variables are expressed as percentages (%). Categorical variables were analysed using the chi-square test. Continuous variables (e.g., age) were analysed using the t test. A logistic regression model was employed to analyse the relationship between constipation and OBS. Both unadjusted and multivariate adjusted models were employed: Model I, without adjusting for any covariates; Model II, with adjustments for sex, age, and race; and Model III, adjusted for covariates in Model II as well as education level, PIR, BMI, diabetes, hypertension, arthritis, heart disease, lung disease, liver disease, and cancer. The dose–response relationship between OBS and the risk of constipation was assessed using restricted cubic spline curves (RCS). In addition, stratified analyses were performed to further validate the robustness of the results. Statistical significance was determined by comparing adjusted odds ratios (ORs) with 1.0 and providing 95% confidence intervals (CIs). All the statistical analyses were performed using R version 4.2.1 (R Foundation for Statistical Computing, Vienna, Austria; http://www.r-project.org), and a two-sided P value < 0.05 indicated statistical significance.

## Results

### Baseline characteristics

A total of 10,742 subjects with complete information were included in the study after combining covariates. Among them, 1,053 were identified as constipated. The mean age of the subjects was 46.94 ± 16.461 years, with 51% being female and the majority being non-Hispanic whites (73.06%). Continuous OBS data underwent quartile transformation, resulting in subgroup determinations labelled Q1, Q2, Q3, and Q4. These quartile ranges for OBS varied for females (0–17, 17–23, 23–28, and 28–39) and males (0–16, 16–22, 22–27, and 27–37). The sample sizes for each OBS subgroup (Q1, Q2, Q3, and Q4) were 2,484, 2,645, 2,470, and 3,143, respectively. The allocation scheme for OBS components is detailed in Table 1. In contrast to the control group, the constipation group exhibited lower education, wealth, BMI, and hypertension incidence, as well as a lower OBS. No significant differences were observed in the remaining covariates (Table 2).

**Table 1:** Baseline characteristics of the NHANES participants (2005–2010)

Characteristic	*N*^1^	Overall, *N* = 10,742 (100%)^2^	Normal, *N* = 9689 (90%)^2^	Constipated, *N* = 1053 (9.5%)^2^	*P *Value^3^
**Age (years)**	10,742	46.94 (16.46)	47.19 (16.38)	44.53 (17.03)	< 0.001
**Sex**	10,742				< 0.001
female		5,389 (51.44%)	4,632 (48.95%)	757 (75.10%)	
male		5,353 (48.56%)	5,057 (51.05%)	296 (24.90%)	
**Race**	10,742				< 0.001
Mexican American		1,833 (7.64%)	1,671 (7.51%)	162 (8.83%)	
Other Hispanic		828 (3.97%)	735 (3.92%)	93 (4.43%)	
Non-Hispanic White		5,630 (73.06%)	5,141 (73.89%)	489 (65.18%)	
Non-Hispanic Black		2,047 (10.36%)	1,769 (9.75%)	278 (16.16%)	
Other Race—Including Multi-Racial		404 (4.97%)	373 (4.92%)	31 (5.41%)	
**Education level**	10,742				< 0.001
< = High school		5,338 (40.67%)	4,723 (39.42%)	615 (52.53%)	
> High school		5,404 (59.33%)	4,966 (60.58%)	438 (47.47%)	
**Family PIR**	10,742				< 0.001
< 2		4,754 (31.74%)	4,184 (30.40%)	570 (44.44%)	
> = 2		5,988 (68.26%)	5,505 (69.60%)	483 (55.56%)	
**BMI**	10,742				< 0.001
under/normal weight		3,017 (31.00%)	2,643 (30.16%)	374 (38.92%)	
overweight		3,705 (33.69%)	3,373 (33.79%)	332 (32.68%)	
obese		4,020 (35.31%)	3,673 (36.04%)	347 (28.41%)	
**Diabetes**	10,742	1,224 (7.81%)	1,099 (7.82%)	125 (7.70%)	0.9
**Hypertension**	10,742	3,790 (30.03%)	3,465 (30.40%)	325 (26.54%)	0.028
**Arthritis**	10,742	3,047 (24.79%)	2,759 (24.87%)	288 (23.96%)	0.6
**Heart disease**	10,742	895 (6.13%)	800 (6.11%)	95 (6.28%)	0.9
**Pulmonary disease**	10,742	1,870 (17.45%)	1,658 (17.06%)	212 (21.16%)	0.029
**Liver disease**	10,742	366 (3.11%)	337 (3.17%)	29 (2.54%)	0.4
**Cancer**	10,742	979 (8.27%)	885 (8.19%)	94 (9.01%)	0.6
**OBS**	10,742	23.02 (6.98)	23.22 (6.92)	21.07 (7.25)	< 0.001
**OBS group**	10,742				< 0.001
Q1		2,484 (19.51%)	2,155 (18.39%)	329 (30.15%)	
Q2		2,645 (22.70%)	2,383 (22.49%)	262 (24.64%)	
Q3		2,470 (24.16%)	2,251 (24.56%)	219 (20.36%)	
Q4		3,143 (33.64%)	2,900 (34.56%)	243 (24.85%)	

^1^N not Missing (unweighted)

^2^Mean (SD) for continuous; n (%) for categorical

^3^Wilcoxon rank-sum test for complex survey samples; chi-squared test with Rao & Scott's second-order correction

**Table 2:** Oxidative balance score assignment scheme

OBS components	Property	Male			Female		
		**0**	**1**	**2**	**0**	**1**	**2**
**Dietary OBS components**							
Dietary fibre (g/d)	A	< 13	13–20.25	≥ 20.25	< 10.90	10.90–16.45	≥ 16.45
β-Carotene (RE/d)	A	< 98.28	98.28–328.69	≥ 328.69	< 99.87	99.87–350.78	≥ 350.78
Vitamin B2 (mg/d)	A	< 1.80	1.80–2.65	≥ 2.65	< 1.40	1.40–2.03	≥ 2.03
Niacin (mg/d)	A	< 21.93	21.93–31.92	≥ 31.92	< 15.92	15.92–22.84	≥ 22.84
Vitamin B6 (mg/d)	A	< 1.68	1.68–2.53	≥ 2.53	< 1.26	1.26–1.86	≥ 1.86
Total folate (mcg/d)	A	< 331.0	331.0–496.5	≥ 496.5	< 260.5	260.5–384.5	≥ 384.5
Vitamin B12 (mcg/d)	A	< 3.86	3.86–6.56	≥ 6.56	< 2.72	2.72–4.76	≥ 4.76
Vitamin C (mg/d)	A	< 44.10	44.10–106.19	≥ 106.19	< 41.75	41.75–92.09	≥ 92.09
Vitamin E (ATE) (mg/d)	A	< 5.54	5.54–8.69	≥ 8.69	< 4.43	4.43–7.04	≥ 7.04
Calcium (mg/d)	A	< 727.00	727.00–1120.92	≥ 1120.92	< 604.02	604.02–917.48	≥ 917.48
Magnesium (mg/d)	A	< 255.5	255.5–357.0	≥ 357.0	< 200.5	200.5–281.5	≥ 281.5
Zinc (mg/d)	A	< 10.09	10.09–14.89	≥ 14.89	< 7.30	7.30–10.61	≥ 10.61
Copper (mg/d)	A	< 1.09	1.09–1.55	≥ 1.55	< 0.88	0.88–1.23	≥ 1.23
Selenium (mcg/d)	A	< 99.35	99.35–140.10	≥ 140.10	< 71.45	71.45–101.60	≥ 101.60
Total fat (g/d)	P	≥ 100.14	67.18–100.14	< 67.18	≥ 72.57	48.90–72.57	< 48.90
Iron (mg/d)	P	≥ 19.31	13.07–19.31	< 13.07	≥ 14.48	9.98–14.48	< 9.98
**Lifestyle OBS components**							
Physical activity (MET-minute/week)	A	< 286	286–2400	≥ 2400	< 115	115–1080	≥ 1080
Alcohol (drinks/d)	P	≥ 30	0–30	None	≥ 15	0–15	None
Body mass index (kg/m2)	P	≥ 30.27	26.00–30.27	< 26.00	≥ 31.44	25.34–31.44	< 25.34
Cotinine (ng/ml)	P	≥ 1.62	0.03–1.62	< 0.03	≥ 0.149	0.020–0.149	< 0.020

The dietary components did not include nutrients obtained from dietary supplements or medications

*OBS* oxidative balance score, *A* antioxidant, *P* prooxidant, *RE* retinol equivalent, *ATE* alpha-tocopherol equivalent, *MET* metabolic equivalent

### Relationship between OBS and constipation

In this study, we explored the relationship between OBS and constipation incidence using three models derived from weighted logistic regression analysis: Model 1, Model 2, and Model 3. The results of these models are summarized in Table 3. Model 3 was specifically chosen for analysis in this paper due to its enhanced stability achieved through adjustments for relevant covariates. According to Model 3, there was a negative association between constipation and OBS. Specifically, each one-unit increase in OBS was associated with a 4% decrease in constipation incidence (OR: 0.96 (0.95, 0.97), *P* < 0.001). Within the OBS subgroup, Model 3 revealed significant negative associations between the risk of constipation and the second, third, and fourth quartiles of OBS compared to the lowest quartile (Q2: 0.72 (0.55, 0.95), *P* = 0.024; Q3: 0.59 (0.47, 0.75), *P* < 0.001; Q4: 0.54 (0.41, 0.70), *P* < 0.001).

**Table 3:** Association between the OBS and constipation prevalence based on weighted logistic regression analysis

Exposures	Model 1		Model 2		Model 3	
	OR [95%CI]	*P*	OR [95%CI]	*P*	OR [95%CI]	*P*
OBS	0.96 [0.95, 0.97]	< 0.001	0.96 [0.95, 0.97]	< 0.001	0.96 [0.95, 0.97]	< 0.001
Q1	Ref	-	Ref	-		-
Q2	0.67 [0.52, 0.87]	0.003	0.69 [0.52, 0.91]	0.01	0.72 [0.55, 0.95]	0.024
Q3	0.51 [0.40, 0.63]	< 0.001	0.54 [0.42, 0.70]	< 0.001	0.59 [0.47, 0.75]	< 0.001
Q4	0.44 [0.35, 0.55]	< 0.001	0.49 [0.38, 0.62]	< 0.001	0.54 [0.41, 0.70]	< 0.001
P for trend	< 0.001		< 0.001		< 0.001	
Dietary OBS	0.95 [0.94, 0.96]	< 0.001	0.95 [0.94, 0.96]	< 0.001	0.96 [0.95, 0.97]	< 0.001
Q1	Ref	-	Ref	-	Ref	-
Q2	0.78 [0.61, 0.99]	0.004	0.74 [0.58, 0.94]	0.017	0.79 [0.62, 1.01]	0.057
Q3	0.51 [0.42, 0.63]	< 0.001	0.56 [0.45, 0.69]	< 0.001	0.62 [0.50, 0.77]	< 0.001
Q4	0.41 [0.33, 0.51]	< 0.001	0.42 [0.33, 0.55]	< 0.001	0.49 [0.39, 0.61]	< 0.001
P for trend	< 0.001		< 0.001		< 0.001	
Life OBS	1.06 [1.00, 1.11]	0.036	1.04 [0.98, 1.10]	0.2	1.02 [0.95, 1.09]	0.6
Q1	Ref	-	Ref	-	Ref	-
Q2	1.00 [0.75, 1.32]	> 0.9	0.94 [0.70, 1.25]	0.7	0.88 [0.66, 1.18]	0.4
Q3	1.14 [0.83, 1.56]	0.4	1.08 [0.79, 1.48]	0.6	0.98 [0.69, 1.40]	> 0.9
Q4	1.20 [0.95, 1.53]	0.12	1.11 [0.87, 1.42]	0.4	0.97 [0.72, 1.32]	0.9
P for trend	0.091		0.262		0.699	

### Relationship between dietary OBS/lifestyle OBS and constipation

The results of the multivariate logistic regression analyses evaluating the relationships between dietary and lifestyle OBS and constipation are presented in Table 3. Similar to total OBS, the risk of constipation demonstrated a negative association with dietary OBS, with a 4% reduction in constipation incidence for each unit increase in the dietary component of OBS (OR: 0.96 (0.95, 0.97), *p* < 0.001). Within the dietary OBS subgroup, model 3 indicated a statistically significant negative association between the risk of constipation and both the third and fourth quartiles of OBS when compared to the lowest quartile (Q3: 0.62 (0.50, 0.77), *P* < 0.001; Q4: 0.49 (0.39, 0.61), *P* < 0.001). However, no significant association was observed between lifestyle OBS and the incidence of constipation (Table 3).

### Stratified analysis of the incidence of OBS and constipation

**Fig. 2:**
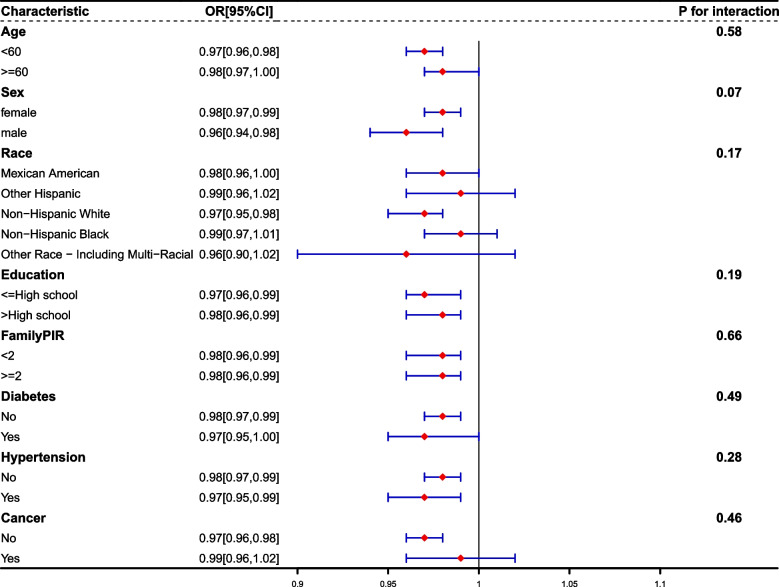
Stratified analysis for the association between OBS and constipation prevalence in US adults, NHANES 2005–2010

In this section, our aim was to investigate the impact of key variables on the relationship between OBS and constipation through stratified analyses. As shown in Fig. 2, the interaction *P* values for all subgroups (age, sex, race, education level, poverty-to-income ratio, diabetes status, hypertension status, and cancer status) were greater than 0.05. This suggests that our findings remained consistent across all subgroups. In the gender subgroups, we found that the effect of OBS on the incidence of constipation was essentially the same in men and women. (Table S1 and S2).

### Analysis of restricted cubic spline regression

**Fig. 3:**
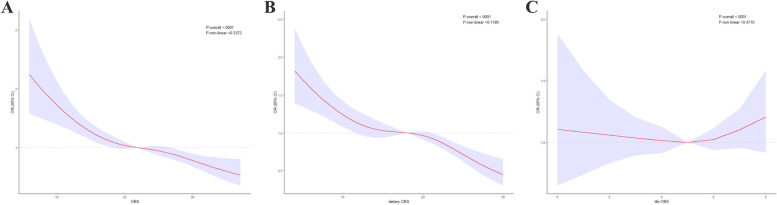
Dose–response associations between OBS, dietary OBS, and lifestyle OBS and risk of constipation. **A** OBS; **B** Dietary OBS; **C** Lifestyle OBS. The solid lines and shaded areas represent the central risk estimates and 95% CIs. There was a linear association of OBS/dietary OBS/lifestyle OBS with risk of depressive symptoms in logistic regression based on restricted cubic splines (*P* for non-linearity = 0.3373; *P* for non-linearity = 0.1190; *P* for non-linearity = 0.4710). Models were adjusted for age, sex, race, education level, PIR, BMI, diabetes, hypertension, arthritis, heart disease, lung disease, liver disease, and cancer; Abbreviation: OBS, oxidative balance score; ORs, odds ratios; CIs, confidence intervals

Through RCS analysis of weighted multivariate logistic regression adjusted for covariates, we discovered a linear relationship (*P*-nonlinear > 0.05) between OBS, the dietary component of OBS, the lifestyle component of OBS, and constipation incidence (Fig. 3A-C). Our findings suggest a negative correlation between OBS and dietary OBS and the incidence of constipation, whereas in lifestyle OBS there was no significant correlation.

## Discussion

To explore the connection between the OBS and constipation, we conducted a cross-sectional analysis involving 10,742 individuals from the NHANES cohort. Our analysis revealed a negative association between the incidence of constipation and both overall OBS and diet-related OBS. However, we observed a generally positive association with lifestyle-related OBS, although it was not statistically significant. These trends were consistent across sex subgroups. In summary, our findings suggest that higher OBSs are linked to a decreased risk of constipation. This finding underscores the importance of maintaining an antioxidant-rich diet for disease prevention, particularly in the case of constipation.

Several dietary factors have been shown to be associated with the development of constipation. A meta-analysis demonstrated the efficacy of fibre supplementation in alleviating constipation [20]. Additionally, Huang et al. identified a potential negative association between niacin intake and constipation incidence, suggesting a nonlinear relationship [21]. In a randomized controlled trial, MgO was found to significantly enhance bowel frequency and quality of life scores, indicating its potential therapeutic benefit for constipation [22]. Moreover, magnesium-based compounds may facilitate stool softening by retaining water in the intestinal lumen [23]. Selenium, an essential micronutrient with various physiological roles, including antioxidant defense and anti-inflammatory effects, has been linked to a reduced risk of chronic constipation [24]. OBS dietary pro-oxidants include fat and iron, with high-fat diets being an important predisposing factor for constipation [25]. Consistently, the dietary OBS was negatively associated with constipation incidence, likely due to the high antioxidant content in the diet, which aligns with our study findings.

In contrast, our study did not reveal a significant association between the lifestyle OBS and constipation incidence. Consistent with our findings, one study failed to establish a strong association between self-reported physical inactivity and fewer than three bowel movements per week or hard/lumpy stools [14]. Furthermore, moderate and light alcohol consumption showed no association or only a weak correlation with gastrointestinal symptoms [26]. Notably, in adults with poor diet quality, exposure to environmental tobacco smoke (ETS) appeared to be positively linked to chronic constipation in terms of stool frequency, while no significant difference was detected in subgroups with healthier eating habits [27]. This finding aligns closely with our results, indicating that none of the lifestyle factors were significantly correlated with the incidence of constipation.

Studies have shown that oxidative stress contributes to constipation by affecting the intestinal flora as well as enteric neurons, and that a reduction in the diversity of intestinal flora as well as enteric neurons can be found in constipated patients. Oxidative stress is evident in constipated animals, as well as in those with colorectal cancer and other chronic diseases linked to constipation, leading to impaired bowel motility [28]. Constipation is significantly negatively correlated with antioxidant activity, suggesting that excessive constipation diminishes the body's antioxidant capacity. Moreover, constipation induces the accumulation of Ca^2+^ in colonic cells, subsequently triggering increased ROS production, thereby reducing cell viability and ultimately resulting in cell death [29]. Furthermore, constipation has been associated with the downregulation of cuprous zinc superoxide dismutase, manganese superoxide dismutase, and catalase, along with the upregulation of nitric oxide synthase and its product NO [30]. Hence, the prevention of free radical production through antioxidant therapy has emerged as a feasible strategy for preventing constipation. Remarkably, our study highlights the significant contribution of antioxidant effects from food as a crucial source of antioxidants in the body.

Our study has several strengths. First, we acquired a large and representative sample of Americans by amalgamating all available consecutive NHANES cycles, enabling us to precisely investigate the relationship between constipation and OBS. Second, we meticulously adjusted for numerous covariates, including income, race, sex, and comorbidities, to bolster the reliability of our findings. Third, we conducted subgroup analyses based on age, sex, race, income, education, and common comorbidities, thereby further corroborating the robustness of our results.

However, there are several limitations to consider. First, the cross-sectional study design complicates the exploration of a causal relationship between oxidative status and constipation, and further research is needed to explore the causal relationship. Second, certain covariates, such as laxative use, were excluded from the analyses due to insufficient data from all volunteers, which leads to the possibility that the results we examined may be subject to some error. Third, the two-day dietary recall in the NHANES database has only two complete days of dietary intake data, leading to some possible discrepancies in the results. Finally, additional validation of the results in non-Western countries is warranted due to variations in diet and lifestyle between Western and non-Western nations.

## Conclusion

In our study, we found that the oxidative balance score (OBS), which provides a comprehensive measure of oxidative stress burden in individuals, was linked to constipation incidence. Specifically, an increase in OBS was associated with a linear decrease in constipation incidence. Thus, OBS, particularly dietary OBS, has emerged as a significant avenue for preventing constipation, highlighting the potential role of dietary modifications in preventing constipation. However, the mechanism by which OBS affects constipation needs to be further investigated.

### Supplementary Information

Supplementary Material 1.

Supplementary Material 2.

## Data Availability

The datasets generated and/or analyzed during the current study are available in the NHANES database, https://wwwn.cdc.gov/nchs/nhanes/.
