# Supplementary material for: Associations between the oxidative balance score and constipation: a cross-sectional study of the NHANES, 2005–2010
Source: BMC Public Health. 2024 Jul 16;24:1908. doi: 10.1186/s12889-024-19428-3 (PMC11253473; doi:10.1186/s12889-024-19428-3)
Supplement: Supplementary file 1 — Supplementary Material 1. [file 12889_2024_19428_MOESM1_ESM.docx]

| Exposures | Model 1 | | Model 2 | | Model 3 | |
| --- | --- | --- | --- | --- | --- | --- |
|  | OR [95%CI] | P | OR [95%CI] | P | OR [95%CI] | P |
| OBS | 0.94 [0.92, 0.96] | <0.001 | 0.94 [0.92, 0.96] | <0.001 | 0.95 [0.93, 0.97] | <0.001 |
| Q1 | Ref | - | Ref | - |  | - |
| Q2 | 0.65 [0.41, 1.02] | 0.06 | 0.67 [0.42, 1.07] | 0.089 | 0.68 [0.42, 1.09] | 0.11 |
| Q3 | 0.43 [0.27, 0.68] | <0.001 | 0.46 [0.28, 0.75] | 0.003 | 0.50 [0.30, 0.82] | 0.008 |
| Q4 | 0.34 [0.22, 0.53] | <0.001 | 0.37 [0.24, 0.57] | <0.001 | 0.41 [0.25, 0.67] | <0.001 |
| P for trend | <0.001 | | <0.001 | | <0.001 | |
| Dietary OBS | 0.93 [0.91, 0.95] | <0.001 | 0.94 [0.92, 0.96] | <0.001 | 0.94 [0.92, 0.96] | <0.001 |
| Q1 | Ref | - | Ref | - | Ref | - |
| Q2 | 0.75 [0.46, 1.22] | 0.2 | 0.79 [0.48, 1.29] | 0.3 | 0.84 [0.53, 1.34] | 0.057 |
| Q3 | 0.45 [0.31, 0.67] | <0.001 | 0.48 [0.32, 0.72] | <0.001 | 0.52 [0.35, 0.79] | 0.003 |
| Q4 | 0.29 [0.18, 0.45] | <0.001 | 0.32 [0.20, 0.49] | <0.001 | 0.37 [0.23, 0.57] | <0.001 |
| P for trend | <0.001 | | <0.001 | | <0.001 | |
| Life OBS | 1.06 [0.96, 1.16] | 0.2 | 1.06 [0.97, 1.17] | 0.2 | 1.10 [0.96, 1.25] | 0.2 |
| Q1 | Ref | - | Ref | - | Ref | - |
| Q2 | 1.30 [0.78, 2.15] | 0.3 | 1.30 [0.78, 2.16] | 0.3 | 1.32 [0.79, 2.22] | 0.3 |
| Q3 | 1.52 [0.80, 2.87] | 0.2 | 1.53 [0.82, 2.88] | 0.2 | 1.60 [0.80, 3.19] | 0.2 |
| Q4 | 1.36 [0.86, 2.16] | 0.2 | 1.41 [0.89, 2.22] | 0.14 | 1.58 [0.87, 2.86] | 0.13 |
| P for trend | 0.197 | | 0.150 | | 0.138 | |

**Table S1 Association between OBS and incidence of constipation based on weighted logistic regression analysis in male.**
