# Supplementary material for: Associations between the oxidative balance score and constipation: a cross-sectional study of the NHANES, 2005–2010
Source: BMC Public Health. 2024 Jul 16;24:1908. doi: 10.1186/s12889-024-19428-3 (PMC11253473; doi:10.1186/s12889-024-19428-3)
Supplement: Supplementary file 2 — Supplementary Material 2. [file 12889_2024_19428_MOESM2_ESM.docx]

**Table S2. Association between OBS and incidence of constipation based on weighted logistic regression analysis in female.**

| Exposures | Model 1 | | Model 2 | | Model 3 | |
| --- | --- | --- | --- | --- | --- | --- |
|  | OR [95%CI] | P | OR [95%CI] | P | OR [95%CI] | P |
| OBS | 0.96 [0.95, 0.97] | <0.001 | 0.96 [0.95, 0.98] | <0.001 | 0.97 [0.96, 0.98] | <0.001 |
| Q1 | Ref | - | Ref | - |  | - |
| Q2 | 0.68 [0.48, 0.95] | 0.026 | 0.70 [0.50, 0.99] | 0.045 | 0.75 [0.53, 1.06] | 0.10 |
| Q3 | 0.56 [0.41, 0.77] | <0.001 | 0.58 [0.42, 0.80] | 0.001 | 0.64 [0.48, 0.86] | 0.005 |
| Q4 | 050 [0.37, 0.67] | <0.001 | 0.53 [0.39, 0.72] | <0.001 | 0.62 [0.47, 0.83] | 0.002 |
| P for trend | <0.001 | | <0.001 | | <0.001 | |
| Dietary OBS | 0.96 [0.94, 0.97] | <0.001 | 0.96 [0.94, 0.97] | <0.001 | 0.97 [0.96, 0.98] | <0.001 |
| Q1 | Ref | - | Ref | - | Ref | - |
| Q2 | 0.72 [0.52, 1.00] | 0.047 | 0.74 [0.53, 1.03] | 0.070 | 0.79 [0.57, 1.10] | 0.1 |
| Q3 | 0.58 [0.43, 0.77] | <0.001 | 0.60 [0.44, 0.81] | 0.001 | 0.68 [0.51, 0.91] | 0.010 |
| Q4 | 0.44[0.33 0.59] | <0.001 | 0.46 [0.34, 0.62] | <0.001 | 0.56 [0.43, 0.73] | <0.001 |
| P for trend | <0.001 | | <0.001 | | <0.001 | |
| Life OBS | 1.01 [0.94, 1.08] | 0.8 | 1.02 [0.96, 1.09] | 0.5 | 1.01 [0.93, 1.09] | 0.8 |
| Q1 | Ref | - | Ref | - | Ref | - |
| Q2 | 0.78 [0.55, 1.11] | 0.2 | 0.80 [0.57, 1.14] | 0.2 | 0.73 [0.52, 1.03] | 0.075 |
| Q3 | 0.88 [0.65, 1.19] | 0.4 | 0.92 [0.68, 1.24] | 0.6 | 0.81 [0.60, 1.10] | 0.2 |
| Q4 | 0.92 [0.69, 1.23] | 0.6 | 0.98 [0.73, 1.32] | 0.9 | 0.84 [0.59, 1.18] | 0.3 |
| P for trend | 0.759 | | 0.877 | | 0.414 | |
